# Acute Anterior Myocardial Infarction Accompanied by Acute Inferior Myocardial Infarction: A Very Rare Coronary Artery Anomaly

**DOI:** 10.1155/2015/347126

**Published:** 2015-06-07

**Authors:** Y. Alsancak, B. Sezenöz, M. Duran, S. Unlu, S. Turkoglu, R. Yalcın

**Affiliations:** ^1^Department of Cardiology, Ataturk Education and Research Hospital, Bilkent, Ankara, Turkey; ^2^Department of Cardiology, Gazi Mustafa Kemal State Hospital, Besevler, Yenimahalle, Turkey; ^3^Department of Cardiology, Medical Faculty, Gazi University, Besevler, Ankara, Turkey

## Abstract

Coronary artery anomalies are rare and mostly silent in clinical practice. First manifestation of this congenital abnormality can be devastating as syncope, acute coronary syndrome, and sudden cardiac death. Herein we report a case with coronary artery anomaly complicated with ST segment myocardial infarction in both inferior and anterior walls simultaneously diagnosed during primary percutaneous coronary intervention.

## 1. Introduction

Coronary artery anomalies (CAA) are a diverse group of congenital disorders whose manifestations and pathophysiological mechanisms are highly variable. Although most of the anomalies have clinically insignificant repercussions, some of them may have devastating impacts. In symptomatic patients, these anomalies manifest themselves as exercise induced angina, syncope, acute coronary syndrome, or in some cases sudden cardiac death. Sudden cardiac death associated with CAA is especially observed in the young [1].

## 2. Case Report

**Figure 1:**
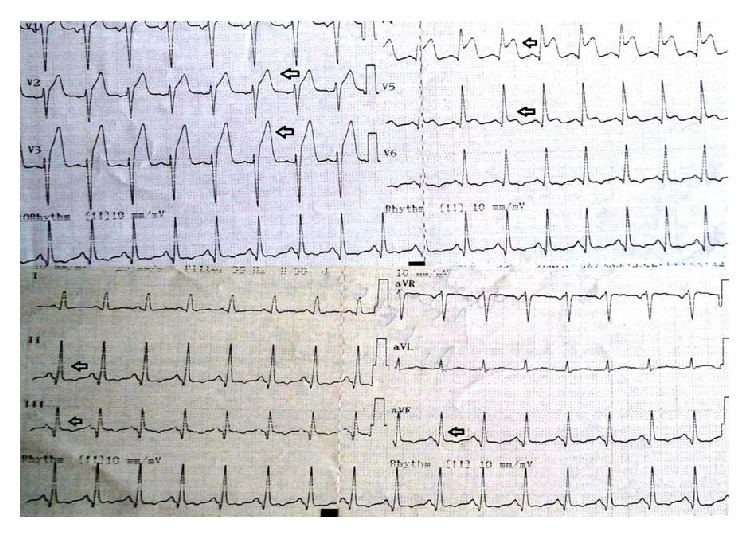
ST segment elevations in precordial leads.

**Figure 2:**
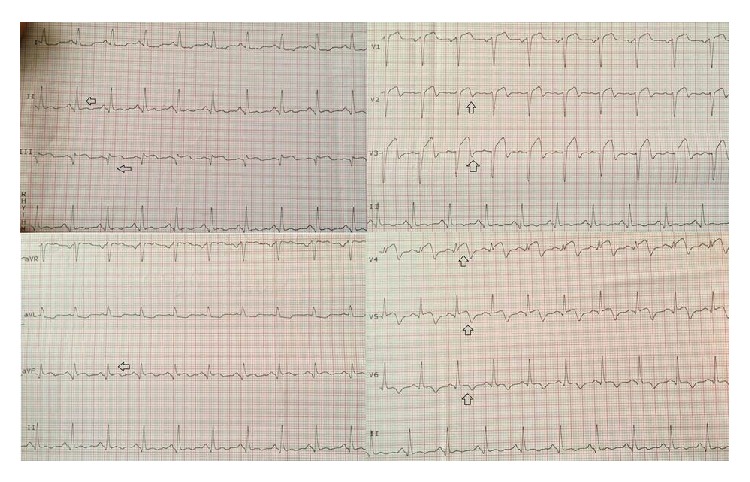
Partial ST segment resolution after fibrinolytic therapy in precordial leads.

**Figure 3:**
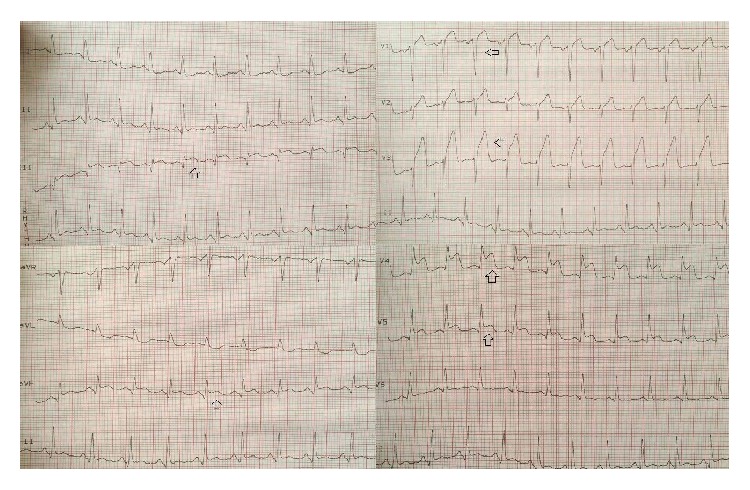
ST segment elevations in both inferior and precordial leads after fibrinolytic therapy.

**Figure 4:**
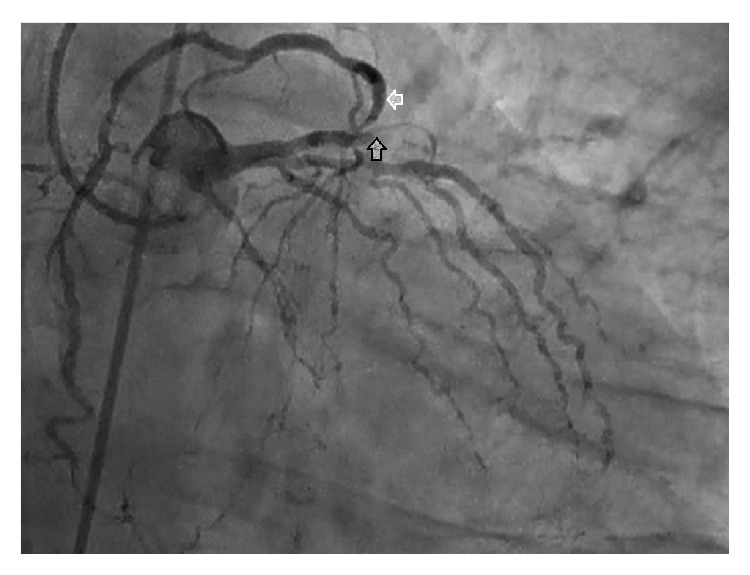
Left anterior descending coronary artery with total occlusion (black arrow) and right coronary artery from proximal segment of left anterior descending coronary artery (white arrow).

**Figure 5 fig5:**
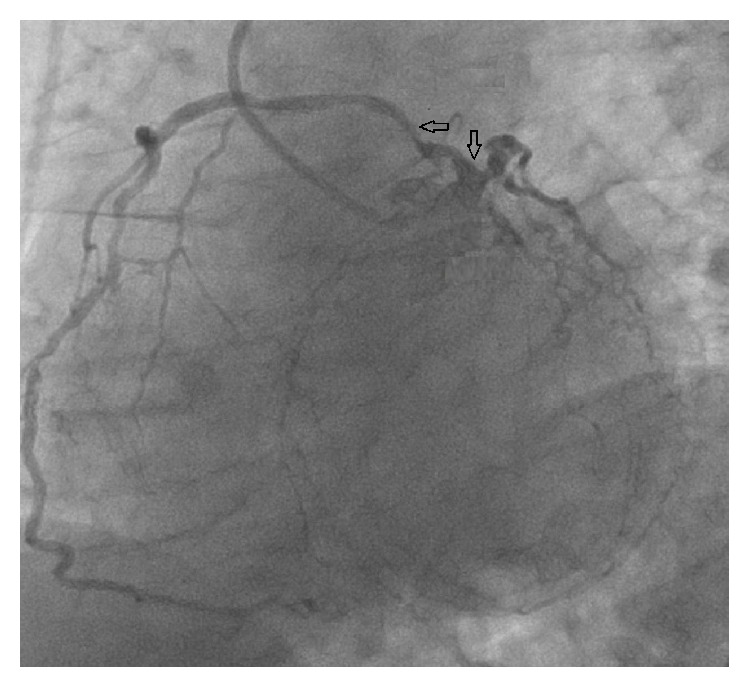
Single coronary ostium and severe ostial lesion of right coronary artery.

**Figure 6 fig6:**
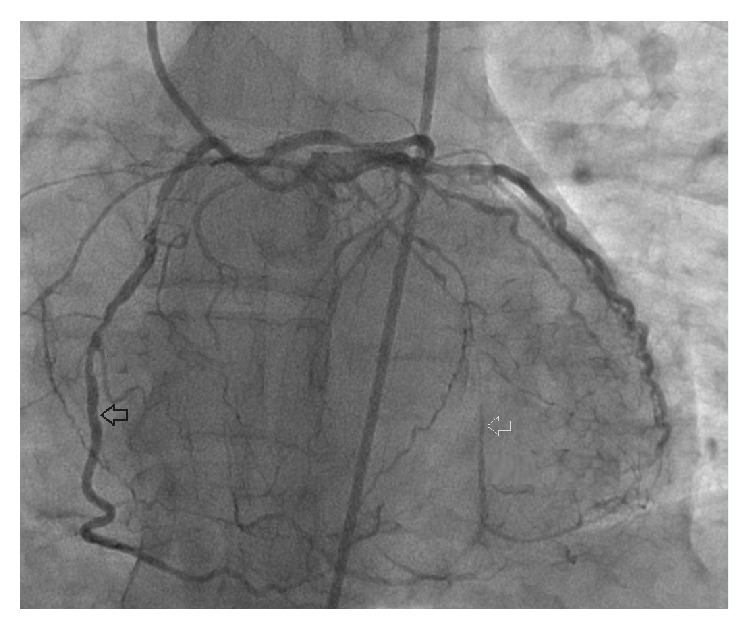
Right coronary artery from proximal left anterior descending coronary artery (black arrow) and retrograde filling of circumflex coronary artery (white arrow).

A 40-year-old male patient was admitted to emergency department with chest pain that had lasted one hour. His electrocardiogram (ECG) on presentation showed ST segment elevations in the precordial leads (Figure 1). Except for smoking there was no reported history of cardiac risk factor. On physical examination his blood pressure was 140/90 mmHg, heart rate 80 beats per minute, and oxygen saturation 96% on room air. His left ventricular functions were found to be depressed during 2D echocardiographic evaluation. After ruling out the possibility of aortic dissection we decided to perform fibrinolytic therapy. After receiving fibrinolytic therapy his chest pain was improved and partial ST segment resolution was observed on his ECG (Figure 2). Troponin level was 0,329 pg/mL. 30 minutes after administration of therapy his chest pain was worsened and his blood pressure dropped to 80/60 mmHg. His control ECGs showed new ST segment elevations in both the inferior and the precordial leads (Figure 3). He was immediately transferred to catheter laboratory for rescue percutaneous coronary intervention (PCI). His coronary angiography (CA) revealed an anomalous right coronary artery (RCA) originating from the proximal portion of left anterior descending (LAD) coronary artery which was totally occluded (Figures 4–7). Also, his CA showed that there was a severe thrombotic occlusion in ostium of RCA and totally occluded circumflex artery (Figure 4). Afterwards, we consulted him about cardiovascular surgery for urgent surgery. Because of his unstable condition and progressive chest pain the patient was deemed an unsuitable candidate for surgery. Under these circumstances we decided to perform PCI. A floppy guide wire was advanced into the RCA and a second wire was reached down to the LAD. During deploying balloon angioplasty to LAD lesion, he developed asystole and subsequently the patient had cardiopulmonary arrest. Despite all our efforts, we lost the patient during cardiac catheterization.

## 3. Discussion

CAA are rare and frequently benign congenital abnormalities of coronary circulation. Although cardiac catheterization is accepted as gold standard for defining CAA, noninvasive multislice computed tomography (CT) plays a substantial role in terms of illustrating the anatomy. Generally, most of the patients were diagnosed incidentally during conventional CA. Coronary artery anomalies have been identified in 0.2% to 1.3% of coronary angiograms and 0.3% in autopsy series [2, 3]. According to a Turkish serial which consisted of 53.655 patients who underwent CA, the estimated incidence of anomalous origin of the RCA from the left sinus of Valsalva or left main coronary artery is 0.1% [4]. Anomalous origin of the RCA from the left sinus of Valsalva traversing between the aorta and pulmonary artery is far more common presentation [5]. Anomalous origin of the RCA from the LAD artery is very rare and frequently benign compared to other types of anomalous origin of the RCA [6]. Sohrabi and his colleagues demonstrated that there was a high prevalence of atherosclerotic coronary artery disease in patients who had an anomalous RCA originating from left coronary sinus [7]. According to a study by Garg and his colleagues, incidence of coronary atherosclerosis in patients with CAA was found to be %10.25 [8]. On the other hand, most of the literatures do not demonstrate a strong relation between CAA and atherosclerosis. Except for a few case reports, myocardial infarction is rarely observed in patients with a history of CAA [9]. Comparing with normal population PCI in patients with CAA has considerable technical difficulties [10]. Postmortem study or CT angiography would have been helpful in order to define the exact course of the coronary artery but unfortunately we did not have the chance to perform any of them. In our case, the patient had a single coronary artery ostium which was occluded by a thrombotic lesion spreading both LAD and ostium of RCA. Even though we did not encounter difficulties during cannulation process, patient's unstable condition and high ischemic burden made our attempt unsuccessful.

## 4. Conclusion

In conclusion, cardiologists should bear in mind that CAA might have vicious outcomes and patients with these anomalies must be evaluated delicately.

## Figures and Tables

**Figure 7:**
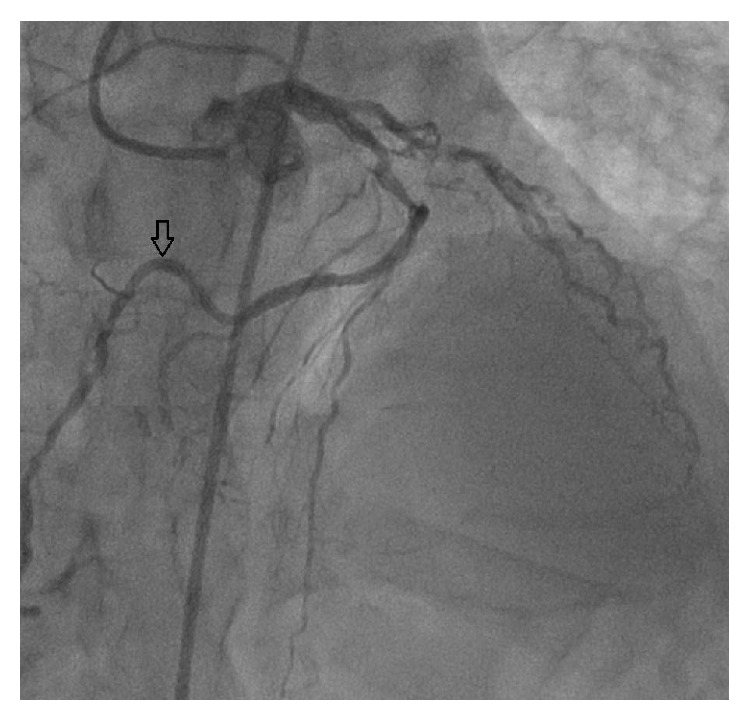
Coronary angiographic appearance of the right coronary artery.
